# Sub-Diffraction Visible Imaging Using Macroscopic Fourier Ptychography and Regularization by Denoising

**DOI:** 10.3390/s18093154

**Published:** 2018-09-18

**Authors:** Zhixin Li, Desheng Wen, Zongxi Song, Gang Liu, Weikang Zhang, Xin Wei

**Affiliations:** 1 Xi’an Institute of Optics and Precision Mechanics, Chinese Academy of Sciences, Xi’an 710119, China; lizhixin2015@opt.cn (Z.L.); ven@opt.ac.cn (D.W.); liugang@opt.cn (G.L.); zhangweikang@opt.cn (W.Z.); weixin@opt.cn (X.W.); 2 University of Chinese Academy of Sciences, Beijing 100049, China

**Keywords:** sub-diffraction visible imaging, computational imaging, Fourier ptychography, phase retrieval, Fourier optics and signal processing

## Abstract

Imaging past the diffraction limit is of significance to an optical system. Fourier ptychography (FP) is a novel coherent imaging technique that can achieve this goal and it is widely used in microscopic imaging. Most phase retrieval algorithms for FP reconstruction are based on Gaussian measurements which cannot extend straightforwardly to long range, sub-diffraction imaging setup because of laser speckle noise corruption. In this work, a new FP reconstruction framework is proposed for macroscopic visible imaging. When compared with existing research, the reweighted amplitude flow algorithm is adopted for better signal modeling, and the Regularization by Denoising (RED) scheme is introduced to reduce the effects of speckle. Experiments demonstrate that the proposed method can obtain state-of-the-art recovered results on both visual and quantitative metrics without increasing computation cost, and it is flexible for real imaging applications.

## 1. Introduction

Improving the resolution of an imaging system is a long-term goal in imaging sciences. It has great importance in many optical implementations and computer vision, including medical imaging, remote sensing, and surveillance. However, in long range imaging, the limited angular extent of the aperture results in low spatial resolution. Several methods have been proposed to prevent resolution loss, and the most direct way is to increase the input aperture by using a large lens. However, it is not an ideal solution to physically increase the lens diameter, which leads to expensive and heavy setups. What is more, many corrective optical devices are required to counteract aberrations in these larger lenses. Super-resolution reconstruction is a kind of computational imaging technique, which can improve the spatial resolution by capturing and processing a collection of low-resolution (LR) images. These LR images are subsampled as well as shifted with subpixel precision. New information is contained in each LR image that can be exploited to obtain a high-resolution (HR) image [1]. Actually, it overcomes the pixel-limited resolutions instead of the diffraction blur. Pixel sampling limits are not as critical to many current imaging applications because current sensor pixels are approaching the diffraction limit of visible light [2]. Synthetic aperture radar (SAR) techniques are also useful ways to improve imaging resolution, which work in long-wavelength regimes. For SAR, full complex filed (amplitude and phase) can be directly recorded since the antenna has picosecond timing resolution. Then, these radar returns could stitch together to achieve a HR image. This method is impossible to visible light imaging, because current camera sensors can only measure the intensity of the optical field and lost all phase information [3].

Recent works in ptychography demonstrate that it can recover phase information by intensity measurements and past the diffraction limit of an optical system. The existing research focus on microscopy that image the thin sample with a smooth phase [4]. It is worth noting that the imaged samples must be thin for Fourier ptychography microscopy (FPM). If this assumption is not satisfied, the LR images at different incident angels cannot uniquely map to different passbands of the spectrum, and the panning spectrum constraint cannot be accurately imposed to recover the HR complex image [5,6]. Dong et al. first extended the ptychography to macroscopic imaging, which recovered a super-resolution image of an object placed at the far field by scanning the entire camera at different x-y positions. However, the imaging samples are real daily objects with “optically rough” surface for long distance imaging system. This leads to strong speckle noise because of coherent illumination. 

In this work, we make two contributions toward sub-diffraction imaging with FP method. First, we show how the reweighted amplitude flow scheme can be applied to Fourier ptychographic reconstruction. Second, we show how the Regularization by Denoising (RED) framework can be plugged into our long range sub-diffraction imaging model. The recovered results are compared with other methods. It shows that our proposed framework offers excellent reconstruction performance under different noise type.

## 2. Related Work

### 2.1. Macroscopic Fourier Ptychography

Fourier ptychography is a newly reported computational imaging technique, which enables imaging past the diffraction limit of optical system. It is essentially a synthetic aperture technology, but it does not require the measurement of phase information. FP illuminates the object with different angels, and correspondingly gets multiple LR images describing different spatial spectrum bands of the object. These captured intensity images have high overlaps ratio between adjacent measurements, which permits the recovery of lost phase information while using reconstruction algorithm. Then, a large field-of-view (FOV) and high resolution image of the object can be obtained by stitching spectrum bands together in Fourier space.

Fourier ptychography macroscopic imaging is first proposed by Dong et al. [6]. They develop a camera-scanning FP platform to displace the angle-varied illumination, which circumvent the thin specimen assumption that is mentioned above and try to extend the FP concept to macroscopic imaging settings. Holloway et al. propose a prototype for macroscopic FP in a reflection imaging geometry and demonstrate it has potential benefits in improving spatial resolution for long distance rough objects [3]. They adopt the alternating projection (AP) algorithm [7,8], which adds constraints alternately in spatial space and Fourier space. Although higher resolution gains can be achieved compared to traditional super-resolution reconstruction algorithms, this method has weak robustness toward noise corruption, especially for laser speckle noise that is caused by the coherence of illumination source. Then stronger regularization and new reconstruction algorithm are required for long range Fourier ptychography macroscopic imaging.

### 2.2. Coherent Illummination and Speckle Phenomena

**Figure 1:**
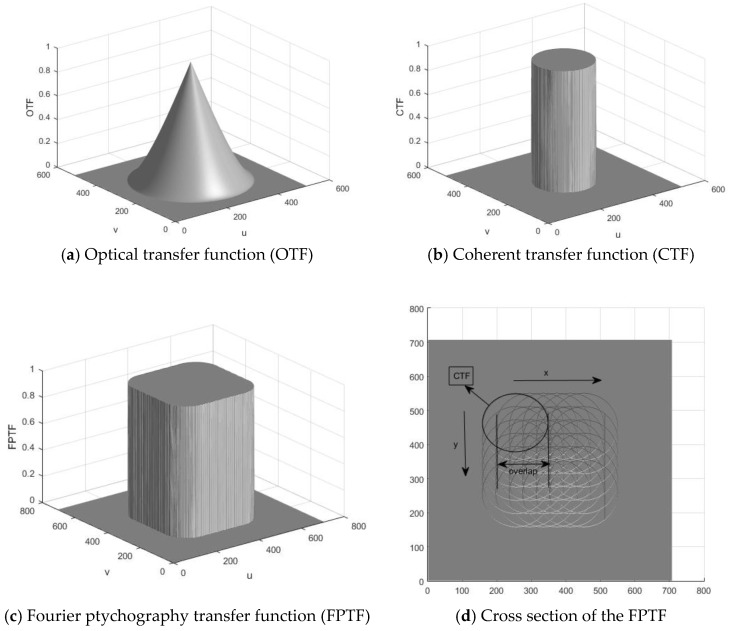
Transfer functions of the imaging system with incoherent illumination, coherent illumination and macroscopic Fourier ptychography, respectively.

When compared with the difference in the transfer functions for incoherent illumination and coherent illumination, the cutoff frequencies of coherent illummination is a half of incoherent illumination if we assume the diffraction-limited system with a circular entrance pupil [9]. Figure 1a shows the optical transfer function (OTF) of an imaging system with incoherent illumination and Figure 1b is the coherent transfer function (CTF) of the imaging system with coherent illumination. It is clear that the CTF can transmits all spatial frequencies within the cutoff frequencies. However, for incoherent imaging system, many important spatial frequencies are attenuated despite higher cutoff frequencies. For the macroscopic Fourier ptychography imaging setup, the CTF is shifted in the Fourier domain by linearly translating the imaging aperture (perpendicular to the optical axis). That is, the Fourier ptychography transfer function (FPTF) is a summation over all shifted CTF, which can synthesize a larger aperture and transmit all spatial frequencies with no attenuation [10].

Most real-word materials are optically rough on the scale of optical wavelength and the surfaces of them fluctuate randomly. The scattering points along the surface act as secondary source with coherent illumination. Laser light scattering from the points interfere with each other that lead to speckle phenomena on the image plane of optical sensor. Actually, speckle is not noise in the conventional sense, which contains the object’s high-resolution information. However, it is recorded by the optical sensor that limits the resolution of coherent imaging system. So, in this work, speckle is regarded as “coherent noise” following the negative exponential distribution [11]. There are two methods to suppress speckle noise, including optical processing and digital image processing [12]. Optical processing methods suppress speckle by reducing spatial coherence of the illumination or diversifying the wavelength and the angle [13], which may affect the reconstruction results of FP. Digital image processing methods are also widely used in SAR and holography to reduce speckle noise, such as median filter method [14], Lee filter [15], nonlocal means algorithm (NLM) [16], and the block matching three-dimensional (3D) algorithm (BM3D) [17,18]. However, most existing research about FP avoid reducing speckle noise by using thin biological samples with smooth phase. Holloway et al. first propose a simple method based on wavelet transformation to suppress laser speckle in long range FP imaging [3]. Removing the speckle from LR images that were captured by macroscopic FP imaging setup requires stronger regularization and more effective reconstruction algorithm.

### 2.3. Phase Retrieval

Phase retrieval algorithms recovering the input signal from only the intensity of the output have become an important role in many modern computational imaging systems [19,20,21]. FP reconstruction can be seen as a typical phase retrieval problem. People can impose the magnitude of a complex filed recorded by image sensors to retrieve the missing phase information and reconstruct the HR image.

From previous work, several algorithms have been proposed to solve this problem. The AP algorithm is simple to use and fast to converge but it is sensitive to measurement noise. It requires long exposure time for capturing high signal-to-noise-ratio (SNR) inputs, which restricts its application range. Bian et al. extend the Wirtinger flow optimization to FP reconstruction [22]. Wirtinger flow optimization for Fourier Ptychography (WFP) minimizes the intensity error between estimated LR images and corresponding measurements while using the gradient scheme and Wirtinger calculus [23,24]. When compared with WFP, the reshaped Wirtinger flow optimization is based on amplitude error that has great advantages in statistical and computational efficiency [25]. The Possion maximum likelihood is proposed for better signal modeling in FPM [26]. However, recent research of Yet et al. demonstrate that the gradient of the Possion log-likelihood function is very similar to amplitude-based cost function [27]. The experiments of Metzler et al. find that it performs slightly worse than amplitude function [28]. Reweighted amplitude flow (RAF) is an amplitude-based phase retrieval algorithm without extra assumption on the signal to be recovered [29]. What is more, it reweights the gradient of loss function in each iteration to obtain the reliable directions pointing to the true value.

## 3. Methods

### 3.1. Image Formation Model

**Figure 2:**
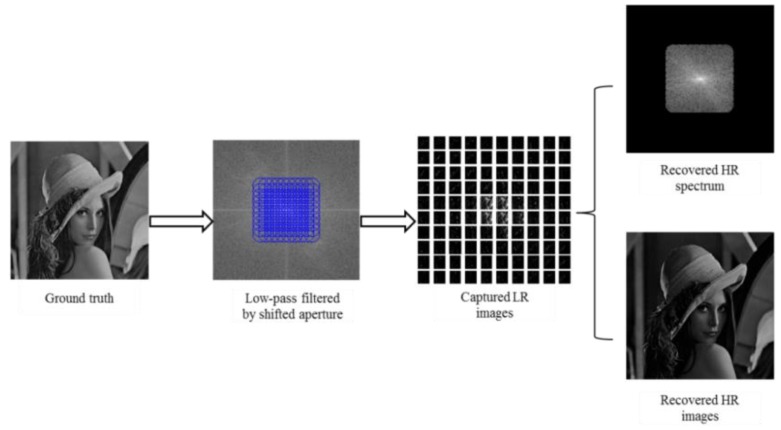
The imaging process of Fourier ptychography.

We first describe the image formation model of the proposed sub-diffraction imaging system. Like other FP methods, a serious of LR images is captured with active illumination. Consider an object that is described by complex reflection function $r\left(x_{0},y_{0}\right)$, where $x_{0}$ and $y_{0}$ are lateral coordinates on the object plane. The limited camera aperture is described by the pupil function $P(x^{\prime },y^{\prime })$, where $x^{\prime }$ and $y^{\prime }$ denote the frequency coordinates in the Fourier plane. For the diffraction-limited setup with a circular entrance pupil, $P(x^{\prime },y^{\prime })$ can be seen as a binary low-pass filter with $P(x^{\prime },y^{\prime })=1$ when the radius is less than or equal to the cutoff frequencies $\xi_{c}$. That is, it can transmit all spatial frequencies within a radius of $\xi_{c}$. In Fourier space, the coherent field spatial distribution is given by

(1) $$U(x^{\prime },y^{\prime })=F\left[\left(r(x_{0},y_{0})\right)\right]P(x^{\prime },y^{\prime }),$$

where F is the Fourier transform operator. To capture a serious of band-limited LR images, the aperture pupil is recentered at m different locations $\left(c_{x^{\prime }}(i),c_{y^{\prime }}(i)\right)$ in the Fourier domian, i=1,2,…m. Since the real imaging sensors can only record (squared) magnitudes of the image pixels and lose phase information, the final image information of Fourier ptychographic setup follows

(2) $$\begin{array}{ll} I(x,y) & ={\left|F^{-1}(U(x^{\prime }-c_{x^{\prime }}(i),y^{\prime }-c_{y^{\prime }}(i)))\right|}^{2}\\ & ={\left|F^{-1}\left\{P(x^{\prime }-c_{x^{\prime }}(i),y^{\prime }-c_{y^{\prime }}(i))F\left[\left(r(x_{0},y_{0})\right)\right]\right\}\right|}^{2} \end{array}$$

where $F^{-1}$ denotes the inverse Fourier operator. It is easy to reconstruct the HR image of object when the complex field $F\left[\left(r(x_{0},y_{0})\right)\right]$ is recovered. The whole imaging process is shown in Figure 2.

### 3.2. Optimization Framework

In this subsection, we introduce the new optimization framework for long range FP reconstruction. We first review the reweighted amplitude flow (RAF) algorithm, then introduce the RAF formulation into macroscopic FP reconstruction, and incorporate the Regularization by Denoising (RED) constraint.

#### 3.2.1. Review of Reweighted Amplitude Flow Algorithm

Reweighted amplitude flow algorithm is a newly reported phase retrieval technique to find an n-dimensional solution x to a system of quadratic equations of the form $y_{i}={\left|\langle a_{i},x\rangle \right|}^{2}$ [29]. Where $a_{i}\in {\mathbb{R}}^{n}$ are feature/sensing vectors, $x\in {\mathbb{R}}^{n}$ is the wanted unknown signal vector, and $y_{i}$ are given observations. Following the least-squares criterion, the problem of solving systems of quadratic equations is recast as a minimization problem

(3) $$L(x)=\frac{1}{2m}\sum_{i=1}^{m}{\left(y_{i}-\left|\langle a_{i},x\rangle \right|\right)}^{2},$$

where *m* is the measurements. The RAF develop a flexible scheme reweighted the gradients of the loss function. x is updated in a gradient descending manner as

(4) $$\begin{array}{ll} x^{t+1} & =x^{t}-\mu^{t}\nabla L_{rw}(x^{t};y_{i})\\ & =x^{t}-\mu^{t}w_{i}^{t}\nabla L(x^{t};y_{i}), \end{array}$$

where $\nabla L_{rw}(x^{t})$ is the reweighted gradient at the current point $x^{t}$, μ is the step size, and ${\left\{w_{i}\right\}}_{1\leq i\leq m}$ are weights.

#### 3.2.2. Reweighted Amplitude Flow for Fourier Ptychography (RAFP)

To introduce the RAF framework into FP reconstruction, we rewrite the formulation of the imaging model (Equation (2)) as

(5) $$y_{i}={\left|A_{i}z\right|}^{2},$$

here $y_{i}$ denotes the LR intensity measurements I(x,y), $A_{i}$ corresponds to a linear transform matrix incorporating the inverse Fourier transform $F^{-1}$ and low-pass filtering of pupil function $P(x^{\prime },y^{\prime })$, and z is the HR spectrum of object. Then, the reconstruction of HR image translates into a standard phase retrieval problem. According to the research [29], we derive the RAF optimization algorithm to solve the above imaging model. The model becomes.

(6) $$\min\;L(z)=\frac{1}{2m}\sum_{i=1}^{m}{\left(\left|A_{i}z\right|-\sqrt{y_{i}}\right)}^{2},$$

similar to Equation (4), it amounts to the update formula

(7) $$\begin{array}{ll} z^{t+1} & =z^{t}-\mu^{t}w_{i}^{t}\nabla L(z)\\ & =z^{t}-\frac{\mu^{t}}{m}\sum_{i=1}^{m}w_{i}^{t}(A_{i}z-\sqrt{y_{i}}\frac{A_{i}z}{\left|A_{i}z\right|})A_{i}^{H}, \end{array}$$

where t is the iterate count, $A_{i}^{H}$ is the transposed-conjugate matrix of $A_{i}$, and the convention $\frac{A_{i}z}{\left|A_{i}z\right|}=0$ is adopted if $A_{i}z=0$.

However, there are two main differences compared to RAF algorithm. One is the setting of initial value and the other is the setting of weight. The root-mean-squared measurements estimator is adopted instead of the spectral initialization. The experiments demonstrate that spectral initialization succeeds for Gaussian measurements [23,25,29,30,31], it fails for FP setup (See [32] for detail). This surprising behavior is also noted in the research [22,33]. Bian et al. adopt the center image of LR images as the initial value for FPM imaging. The root-mean-squared measurements are employed as an initial estimator for the proposed method. Experiments show that it is slightly better than the center image. Next, we discuss the setting of weight for gradients.

**Figure 3:**
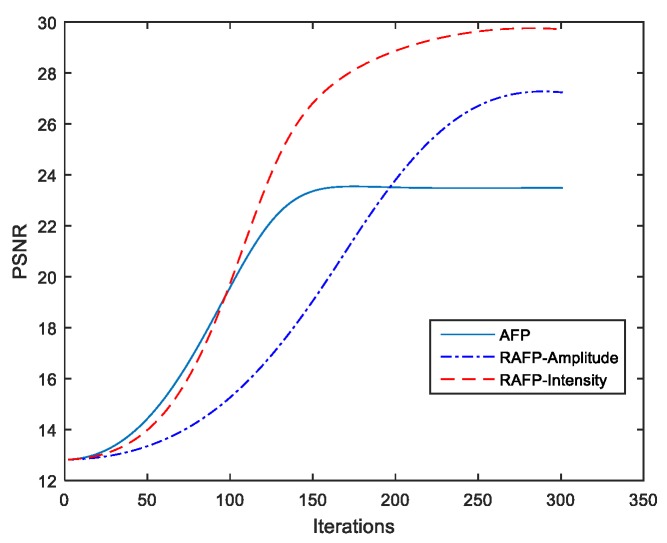
Peak signal to noise ratio (PSNR) for Reweighted Amplitude Flow for Fourier Ptychography (RAFP) algorithm with different weights.

Assuming that ϕ is the wanted unknown signal vector and *z* is the recovered value for it. Unfortunately, the gradient of average in Equation (7) may not point towards true ϕ, when the current iterate value z is not very close to ϕ. Worse still, the summands $(A_{i}z-\sqrt{y_{i}}\frac{A_{i}z}{\left|A_{i}z\right|})A_{i}^{H}$ could give rise to misleading search directions due to the erroneously estimated signs $\frac{A_{i}z}{\left|A_{i}z\right|}\neq \frac{A_{i}\phi }{\left|A_{i}\phi \right|}$, which may drag z away from ϕ. To address this challenge, the RAFP algorithm introduces suitable weights into the Fourier ptychography framework. The weight at the current point $z^{t}$ is given as

(8) $$w_{i}=\frac{{\left|A_{i}z\right|}^{2}/y_{i}}{{\left|A_{i}z\right|}^{2}/y_{i}+\beta_{i}},$$

where ${\left\{\beta_{i}\right\}}_{i=1}^{m}$ are some pre-selected parameters, $y_{i}$ is the intensity measurement, and ${\left|A_{i}z\right|}^{2}$ is recovered value for it. The ratio ${\left|A_{i}z\right|}^{2}/y_{i}$ can be seen as a confidence score on the reliability of corresponding gradient and satisfy ${\left|A_{i}z\right|}^{2}/y_{i}\leq 1$ because of the existence of noise. The larger the confidence score ${\left|A_{i}z\right|}^{2}/y_{i}$, the more similar between the measurements and recovered value. This is a simple way to achieve small weights to the spurious gradients, and large weights to the reliable ones. Thus, the Equation (7) may point to the true value with large probability. In this work, the intensity-based weights are adopted instead of amplitude-based weights to avoid the effect of phase noise on the gradient direction. Figure 3 shows the comparison of peak signal to noise ratio (PSNR) for the RAFP algorithm with different weights. It is clear that the intensity-based weights improve the PSNR about 7dB when compared with no weights for gradients.

#### 3.2.3. Fourier Ptychography via Regularization by Denoising

Here we show how the Regularization by Denoising (RED) [34] framework can be adapted to Fourier ptychography. RED is an efficient approach to solve imaging inverse-problems, which can incorporate an arbitrary denoiser to regularize an arbitrary imaging inverse-problem. It uses the denoising engine in defining the regularization of inverse-problem, making the overall objective function clearer and better defined. To apply RED to Fourier ptychography, we construct an energy function for the proposed imaging model of the form

(9)  E(z)=L(z)+R(z),

where L(z) is a data-fidelity term and R(z) is a regular term. The data-fidelity term L(z) follows the form of Equation (6), which encourages |Az| to match the capture images $\sqrt{y}$. From the research [34], the RED framework defines the regular term R(z), as

(10) $$R(z)=\frac{1}{2}z^{H}[z-f(z)].$$

In this expression, f(z) is the denoising engine that one can choose any filter to plug in the regularization term. Observing the Equation (10), we note that this regularizer not only penalizes the residual difference between recovered value and its denoised self, but it also prevents f(z) from removing structure from z [28]. Then, the energy function of FP reconstruction with RED framework can be described as

(11) $$\arg\;\min\;E(z)=\frac{1}{2m}\sum_{i=1}^{m}{\left(\left|A_{i}z\right|-\sqrt{y_{i}}\right)}^{2}+\frac{\lambda }{2}z^{H}[z-f(z)],$$

here are several methods to solve this problem, including gradient decent method, ADMM method, and fixed-point strategy. Given the gradient of the energy function E(x), the gradient decent method is the simplest option that can be adopted. The gradient of Equation (11) is readily available by

(12) ∇E(z)=w∇L(z)+∇R(z),

where the first term is the reweighted gradient of the data-fidelity term and the gradient of regular term is ∇R(z)=λ(z−f(z)). (See [34] for detail.) Then, the final update formula is

(13) $$\begin{array}{ll} z^{t+1} & =z^{t}-\mu^{t}\nabla E(z)\\ & =z^{t}-\mu^{t}\left[\frac{1}{m}\sum_{i=1}^{m}w_{i}(A_{i}z-\sqrt{y_{i}}\frac{A_{i}z}{\left|A_{i}z\right|})A_{i}^{H}+\lambda^{t}(z-f(z))\right] \end{array}$$

The whole imaging reconstruction framework can be readily summarized in Algorithm 1.

**Algorithm 1** The imaging reconstruction framework**Input:** Captured LR images ${\left\{y_{i}\right\}}_{i=1}^{m}$; sampling matrix ${\left\{A_{i}\right\}}_{i=1}^{m}$.**Output:** Recovered spectrum z.1: **Parameters:** Maximum number of iterations T; step size μ;weighting parameters $\beta_{i}=0.8$; Regularization parameter λ=0.01.2: **Initialization:**
$z^{0}=F(\frac{1}{m}\sum_{i=1}^{m}\sqrt{y_{i}})$.3: **Loop: for**
t=0
**to**
T−1$z^{t+1}\;=\;z^{t}-\mu^{t}\left[\frac{1}{m}\sum_{i=1}^{m}w_{i}(A_{i}z-\sqrt{y_{i}}\frac{A_{i}z}{\left|A_{i}z\right|})A_{i}^{H}+\lambda^{t}(z-f(z))\right]$,where $w_{i}=\frac{{\left|A_{i}z\right|}^{2}/y_{i}}{{\left|A_{i}z\right|}^{2}/y_{i}+\beta_{i}}$ for all 1≤i≤m.4: **end**

## 4. Experiments and Results

In this section, a serious of experiments is conducted to demonstrate the performance of the proposed algorithm.

### 4.1. Numerical Simulation

We now test the efficacy of the proposed method via a number of simulated images reconstruction experiments. In the simulation experiments, the parameters of our sub-imaging setup are set as follows: The illumination wavelength is λ=632.8 nm, the aperture diameter is D=2.5 mm, the focal length of the lens is f=75 mm, the number of vertical and horizontal measurements are m=15×15, and the overlap ratio between two adjacent positions is 65%. We assume that the object (512×512 pixels ‘Lena’ image) itself is 6 cm×6 cm and is located d=5 meters away from the camera. Then, the cutoff frequency of the coherent transfer function (CTF) is $D/\left(\lambda \times d\right)\approx 395\;m^{-1}$, badly blurring out the important features of object. The simulated low-resolution images are obtained according to Equation (3).

Gaussian noise and speckle noise are introduced to simulate the noise corruption. Adding Gaussian noise to the captured LR images is easy. However, speckle is because the underlying random phase of object distorts the intensity field, which is actually not noise. To acquire intensity images with speckle-like features, a Gaussian random surface is simulated for the object. Following previous research [35,36], we change the height function of surface to introduce rapidly-changed random phase for the object. Then, the Equation (2) is utilized to sample the object to obtain LR images with “speckle noise”.

**Figure 4:**
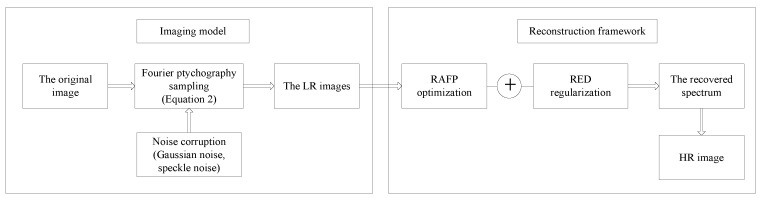
Block scheme of the simulation experiments.

The pipeline of numerical simulation experiments is described in Figure 4.

### 4.2. Criterion

In addition to visual results, two objective quantitative indexes are used in the experiments including peak signal to noise ratio (PSNR) and structure similarity (SSIM). They are widely adopted to assess the quality of processed images compared to benchmark. The PSNR describes the intensity difference between recovered image and the ground truth, and is greater for higher quality image. The SSIM measures the spatial structural closeness between two images, and it is lower when two images are of less similar structural information [37].

### 4.3. Results

First, different denoisers f(z) are applied to the RED framework to study its effect on the algorithm’s performance. Here, the simulated captured images are corrupted with Gaussian noise and laser speckle noise, respectively. The first one is common noise in real imaging implementations that is mostly caused by photoelectric effect and dark current. Laser speckle noise is caused by the light’s spatial-temporal coherence of the illumination source and we simulate this noise by imaging an optically rough object, which means introducing a random phase during the imaging process. Actually, speckle noise is the main noise toward a long distance sub-diffraction imaging system while using Fourier ptychography [3]. Specially, the standard derivation σ of the Gaussian noise ranges from 0.001 to 0.005 and the noise strength α of speckle noise ranges from 1 to 5. Four different denoisers are plugged into the proposed scheme, including median filter, wavelet filter, Lee filter, and BM3D method. Table 1 shows the quantitative results of the proposed method with varying amounts of Gaussian noise. All denoisers produce similar reconstruction results. We can see that even the simplest median filter acts as a reasonable regularizer to our reconstruction problem. BM3D is slightly better than other denoising engine on both PSNR and SSIM. Table 2 shows the quantitative results of the proposed method with varying amounts of speckle noise. The RED significantly improves the performance of reconstruction results as compared to without this regular term. The BM3D improves the PSNR about 10dB and improve the SSIM about 0.2. What’s more, it performs strong robustness especially at high noise strength. This is because BM3D integrates the advantages of the transform domain denoising method and the local average method, which can achieve state-of-the-art denoising performance in terms of both PSNR and SSIM quality. In a word, the RED framework can efficiently suppress different types of noise by plugging a reasonable denoise engine. This regularizer can not only penalize the residual difference between recovered image and denoised self, but also penalize correlations between recovered image and the residual (Equation (10)).

Then, we compare the proposed method (RAFP with RED) against alternating projection (AP) [7,8], Wirtinger flow optimization for Fourier Ptychography (WFP) [22] and truncated amplitude flow (TAF) [31]. AP is a baseline algorithm for Fourier ptychographic reconstruction, which is also the only one algorithm used for long distance sub-diffraction imaging. WFP and TAF are both new phase retrieval algorithms. The Wirtinger flow optimization is based on intensity measurements, while the TAF optimization is based on amplitude measurements. Because the gradient of the Possion log-likelihood function is very similar to amplitude-based cost function [27,28], PWFP [33] is not included in the comparison algorithms. Toward the proposed algorithm, Median and BM3D are used as the denoisers, respectively (RAFP-median and RAFP-BM3D).

**Figure 5:**
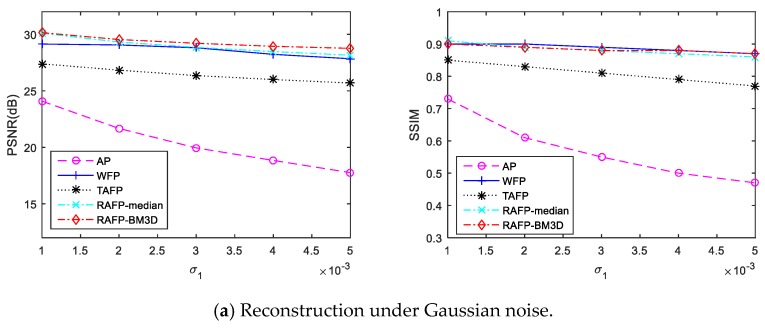

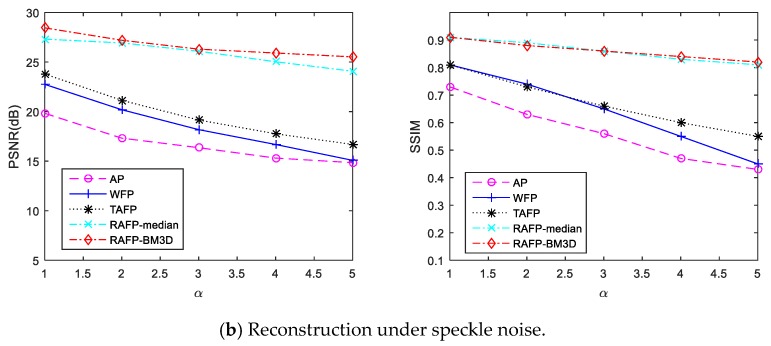
Quantitative comparison of the reconstruction results by different methods under Gaussian noise and speckle noise.

Figure 5a,b show the quantitative reconstruction results by above methods under Gaussian noise and speckle noise, respectively. It is clear that AP algorithm degrades significantly with the increase of noise. From the Figure 5a, WFP performs strong robustness under Gaussian noise corruption, which can achieve reliable results even at high noise levels. From the Figure 5b, the WFP performs not well under speckle noise because of its Gaussian noise assumption. Thus, they can not recognize the multiplicative noise and filter out them. Actually, TAF can be seen as a special case of the reweighted amplitude flow (RAF) algorithm with particular choices of weights $w_{i}$ (when they take 0/1 values). Therefore, its performance is worse than our proposed method. From the results, it can be seen that the proposed algorithm outperforms state-of-the-arts a lot because incorporates the advantages of RAFP and RED framework. It not only reweights the gradients of loss function to reconstruct the true solution with high probability but also recognizes and filters the outliers using RED framework. When compared to the RAFP-BM3D and RAFP-median, filter selection does not have a significant impact on quantitative results, which reduces the complexity of our reconstruction model.

The running time of each method is displayed in Table 3. All the algorithms are implemented using MATLAB R2015a on an Intel Xeon 1.9 GHz CPU computer, with 16GB RAM and 64bit Win7 system. For computational imaging technology, the spatial resolution is increased at the expense of relatively high computation cost. FP reconstruction also requires a large amount of calculation, which limits its application scope to a certain extent, especially for long-distance real imaging implementation. From the Table 3, the proposed methods (RAFP-median and RAFP-BM3D) converge faster than WFP and TAFP, but needs more time consuming than AP. The RAFP-BM3D achieves the best reconstruction results but it requires the most running time. However, the RAFP-median can also achieve satisfactory results and cost relatively low runtime, which may have a wider application.

**Figure 6:**
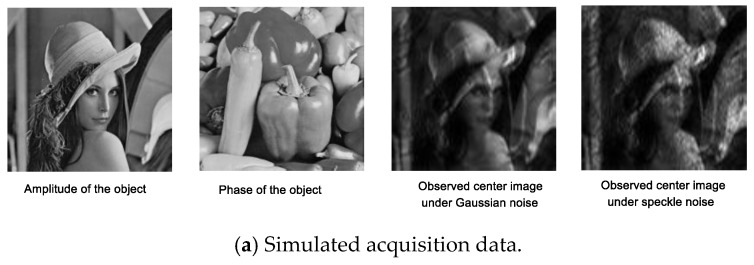

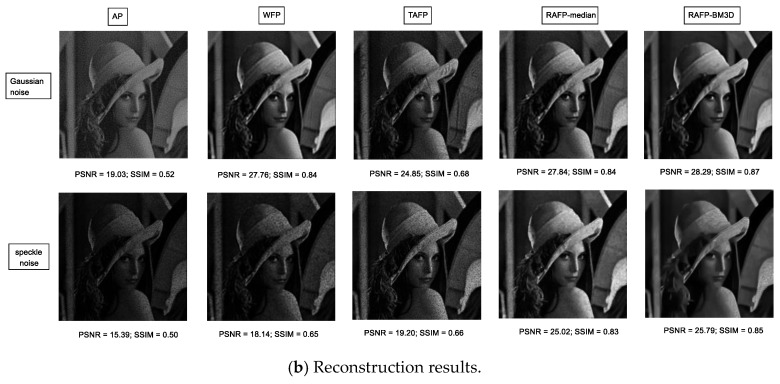
Visual comparison of the reconstruction results by different methods under Gaussian noise and speckle noise.

Figure 6 shows the visual comparison of different methods. Here, the complex object is used to validate the performance of above algorithms. Specifically, the Gaussian noise level is fixed at σ=0.004, and the speckle noise level is fixed at α=3. “Lena” image and “Peppers” image are used as the object’s amplitude and phase image, respectively. The captured LR images have very low SNR due to noise corruption in Figure 6a. Figure 6b describes the visual results among above methods under Gaussian noise and speckle noise, which achieves the same reconstruction performance as before. The reconstruction results of AP are very noisy, although the required running time is the shortest. WFP reconstruction algorithm is based on Gaussian assumption that leads to satisfied results under Gaussian noise corruption. However, it is not effective enough to recognize speckle noise and filter them. When compared to WFP, the TAFP can better preserve image details but there exist serious artifacts. The proposed method outperforms the other three methods by combining reconstruction and filtering jointly. It can achieve state-of-the-art visual results, even with the simplest median filter. Obviously, the RAFP-BM3D can better remove noise, which is at the expense of high computation cost.

## 5. Discussion and Conclusions

In this work, a new FP reconstruction method is proposed for sub-diffraction imaging that can image beyond the diffraction-limit of an optical system. Firstly, the RAF scheme is applied to FP. It reweights the gradients of loss function and offers reliable directions pointing to true value with high probability. Secondly, the RED framework is adapted to our reweighted amplitude-based loss function. It can reduce the noise during recovery process by plugging arbitrary denoiser into the regular term. Furthermore, we can choose the corresponding denoiser according to noise type in measurements, which is very flexible. The performance of our framework is compared to three existing algorithms, including AP, WFF, and TAFP. The experimental results show that the proposed method not only promotes the PSNR and SSIM of the recovered image, but also has strong robustness toward different noise. Especially for real sub-imaging implementations, the main noise corruption is laser speckle noise instead of Gaussian noise. The proposed method outperforms other algorithms and achieves the best results that could be widely used in FP reconstruction.

The choice of denoiser greatly affects the running time of the proposed method. As state-of-the-art filter, the BM3D requires the most running time cost while achieving the best results. From Section 4, it can be seen that the performance of RAFP-median is slightly worse than RAFP-BM3D, but it saves a lot of running time. People can choose the suitable denoiser according to the system’s demand for time and precision, which is very flexible and convenient in practical applications. However, compared to the AP algorithm, the proposed method is still limited in running efficiency. Therefore, shortening the running time of the proposed method is our future work. The parallel computation techniques are employed to accelerate the proposed method.

## Figures and Tables

**Table 1:** PSNR(dB) and structure similarity (SSIM) of the proposed algorithm with different denoisers and varying amounts of Gaussian noise.

	σ=0.001		σ=0.002		σ=0.003		σ=0.004		σ=0.005	
	PSNR	SSIM	PSNR	SSIM	PSNR	SSIM	PSNR	SSIM	PSNR	SSIM
Without RED	29.72	0.90	28.93	0.88	28.43	0.87	28.10	0.86	27.86	0.85
RED-median	30.10	**0.91**	29.32	**0.89**	28.85	**0.88**	28.47	0.87	28.15	0.86
RED-wavelet	30.12	0.90	28.98	0.88	28.57	0.87	28.18	0.86	27.92	0.85
RED-BM3D	**30.15**	0.90	**29.54**	**0.89**	**29.21**	**0.88**	**28.94**	**0.88**	**28.76**	**0.87**

**Table 2:** PSNR(dB) and SSIM of the proposed algorithm with different denoisers and varying amounts of speckle noise.

	α=1		α=2		α=3		α=4		α=5	
	PSNR	SSIM	PSNR	SSIM	PSNR	SSIM	PSNR	SSIM	PSNR	SSIM
Without RED	24.08	0.82	21.36	0.74	19.20	0.66	17.78	0.60	16.70	0.54
RED-median	27.31	**0.91**	26.93	**0.89**	26.09	**0.86**	25.03	0.83	24.07	0.81
RED-Lee filter	26.93	0.89	26. 69	0.88	25.38	0.84	24.46	0.81	23.52	0.78
RED-BM3D	**28.45**	**0.91**	**27.25**	0.88	**26.30**	**0.86**	**25.91**	**0.84**	**25.52**	**0.82**

**Table 3:** Comparison of running time between different algorithms.

	AP	WFP	TAFP	RAFP-median	RAFP-BM3D
Iteration	100	350	300	200	140
Running time(s)	25	332	294	206	630
